# The Art of Learning Empathy: Advancing Organizational Ethics Education With the *SpaceJam* Pedagogical Strategy

**DOI:** 10.1177/10525629261421590

**Published:** 2026-03-06

**Authors:** Joé T. Martineau, Audrey-Anne Cyr

**Affiliations:** 1 Department of Management, HEC Montreal, QC, Canada; 2 Department of Entrepreneurship and Innovation, HEC Montreal, QC, Canada

**Keywords:** organizational ethics education, empathy, pedagogical strategy, arts-based approaches, experiential learning

## Abstract

Amidst the ethical challenges facing the corporate world, there is an urgent demand for enhanced ethics education in business schools. This article introduces *SpaceJam*, an innovative pedagogical strategy designed to nurture empathy—an indispensable capacity for guiding ethical decision-making and behavior within organizational settings. Grounded in a pragmatist and humanistic approach to ethics education, SpaceJam offers a holistic strategy that targets the affective, cognitive, and motivational dimensions of empathy as well as ethical reflection. By diversifying learning spaces—encompassing the classroom, home, and community—and employing experiential and art-based methods, this strategy fosters multiple immersive learning experiences and opportunities in diverse contexts. The core of its integrative nature is manifested through the creation of an artistic portfolio, serving as a fourth learning space for students to express, comprehend, and reflect upon their learning journey through creative work. Implemented on three cohorts of students enrolled in a Bachelor of Business Administration program, the strategy was found effective in cultivating empathy and ethical reflection among future business leaders, offering a promising pathway for business schools to meet the imperative need for ethical education.

## Introduction

For over two decades, ethics education has increasingly become an important component of business school curricula, although the extent and depth of its integration varies significantly across institutions (Nicholson & DeMoss, 2009; Rutherford et al., 2012). This development has largely been driven by the need to address widespread scandals involving fraud, corruption, exploitation, environmental negligence, and various ethical failures among executives and employees worldwide (Crane, 2004; Datar et al., 2011; Swanson, 2004). Indeed, ethics education can contribute to prevent unethical behaviors and strengthen students’ ethical awareness and competencies for ethical reflection and judgment (Lau, 2010). These competencies are crucial for navigating contemporary challenges, including the growing polarization dynamics that increasingly complicate social interactions and decision-making within universities, organizations, and society at large (Fancher, 2021; Martineau & Cyr, 2025; Rekker, 2021).

While ethics education is undoubtedly important, it also faces particular challenges within business schools. Research indicates that a significant proportion of undergraduate business students show limited interest in ethics. For example, Tormo-Carbó et al. (2019) highlight a general lack of engagement with ethical content in business curricula, while Giacalone and Promislo (2013) note that many students enter business programs already predisposed to resist or even dismiss ethical ideals, having been shaped by a materialistic worldview that elevates success, competition, and financial gain over moral considerations. This is especially concerning given that socialization into the business environment and discourse may further stigmatize prosocial qualities and behaviors—such as goodness, compassion, empathy, benevolence, and caring (Giacalone & Promislo, 2013; Holt & Marques, 2012). These circumstances present significant challenges for ethics education in business schools, potentially hindering the development of ethical skills and behaviors in future managers (Cartabuke et al., 2019).

To address these challenges, calls have been made for business and organizational ethics educators to pioneer innovative pedagogical approaches that humanize organizations by nurturing soft skills such as empathy and care (Cohen, 2012; Holt & Marques, 2012; Nussbaum, 2010). These calls emphasize that ethics education should guide students in the meaningful exploration of ethical values and aspirations within organizations, such as integrity, transparency, and commitment to sustainability and social responsibility. Achieving such objectives, however, requires a paradigm shift in the way ethics is taught. It demands a transformation in pedagogical strategies currently predominant in business schools, which mainly emphasize theoretical frameworks, normative principles, and behavioral ethics content (De Los Reyes et al., 2017). Organizational ethics education must also prioritize the cultivation of interpersonal and ethical competencies, such as empathy, ethical reflection, and pro-social behaviors, thus providing students with opportunities not only to learn ethics but also to “live ethics” (Racine et al., 2024). Nevertheless, there is limited literature on the pedagogical approaches that should be employed and their effectiveness in cultivating students’ ethical skills, especially regarding the development of empathy.

In response to these calls, we have developed an integrative pedagogical strategy aimed at addressing the challenges of organizational ethics education (Starkey & Tempest, 2009) by emphasizing experiential and human-centered approaches. Rooted in a humanistic and pragmatist perspective of organizational ethics education (de Colle et al., 2024; Dewey, 1997; Nussbaum, 2010; Pirson, 2020), this strategy—which we term SpaceJam (the Space-crossing Pedagogical Approach to Cognizing Empathy through the Joining of Arts and Materiality)—emphasizes the embodiment of ethics in everyday practice, shifting the focus from learning about ethics to living ethics (Racine et al., 2024; Solberg et al., 1995). Indeed, the SpaceJam strategy views ethics as an interactive, dynamic, contextual, and ongoing process, with empathy playing a crucial role in facilitating the understanding of diverse emotions, ethical values, and behaviors. It integrates a range of experiential and art-based methods across diverse learning spaces, aligning each space’s social and material characteristics (i.e., the classroom, the home, and the community) to expand opportunities for ethical learning and the development of multi-faceted empathy (i.e., cognitive, affective, and motivational). It seeks to cultivate ethical reflection throughout the learning journey by connecting these spaces in a coherent, immersive experience. At the core of this pedagogical strategy is the creation of an artistic portfolio, which serves as a dedicated space for integrating learning and reflecting on experiences across all learning spaces.

In this paper, we intend to:

1. Introduce SpaceJam, an integrative pedagogical strategy, and articulate its theoretical underpinning.
2. Present empirical findings showing how the strategy supports the integration of empathy and ethical reflection in students’ individual ethics and everyday behaviors.

For these purposes, we first review the literature on empathy and organizational ethics education. Following this, we delve into the SpaceJam strategy, beginning with a discussion on its theoretical foundation and its integrative approach to organizational ethics pedagogy. We then describe the context in which the strategy was designed, implemented, documented, and refined in an undergraduate senior course on organizational ethics at a Canadian business school over 3 years and three cohorts of students. Next, we introduce the methods used to assess its effectiveness and examine how students perceived the associated learning opportunities and outcomes. We present empirical evidence illustrating its effects on students’ learning outcomes, showing that the strategy is effective in fostering the development of all three dimensions of empathy in students (affective, cognitive, and motivational), while also encouraging meaningful engagement and reflection on personal and organizational ethics in various social contexts. Finally, we conclude by exploring how the strategy serves as a means for integrating diverse cognitive, affective, and motivational learning methods, while presenting its potential transferability to other pedagogical contexts.

## Teaching Ethics in Business Schools

Business schools offer distinctive contexts in which ethics education targets specific goals. From the literature, we can identify five primary objectives of organizational ethics education: (1) helping students understand that ethics is part of everyday organizations and decision-making (Sims & Felton, 2006); (2) introducing students to theoretical knowledge about ethics (Felton & Sims, 2005); (3) sensitizing students to organizational and governance best practices to prevent unethical behavior (Luthar & Karri, 2005); (4) helping students identify their core values (Sims & Felton, 2006); and (5) promoting the development of students’ individual skills like ethical awareness, sensitivity, reasoning, and empathy, which foster pro-social and ethical behaviors in organizations (Lau, 2010; Martineau et al., 2020). As highlighted in the introduction, it is this ultimate objective of fostering ethical behavior that poses the greatest challenge. Indeed, business school students undergo socialization throughout their academic journey, encountering economic and individualistic self-promotion discourses (Parker, 2018) that can stigmatize pro-social behaviors like empathy (Giacalone & Promislo, 2013; Petriglieri & Petriglieri, 2015).

Traditional approaches to organizational ethics education are typically focused either on the normative or behavioral aspects of ethics in the context of business higher education (De Los Reyes et al., 2017). However, these methods often fall short in adequately preparing students to engage with and respond to the complexities of today’s world. On the one side, normative teaching approaches prioritize critical thinking, open inquiry, analytical methods mobilizing moral philosophies, to stimulate intellectual growth and deeper understanding of ethical issues (DeTienne et al., 2021). Favoring dialogical approaches, such as the Socratic method (Morrell, 2004), normative approaches aim at developing critical thinking abilities and a capacity for differentiated practical reasoning. On the other side, behavioral teaching approaches focus on approaches mobilizing theories and findings from social sciences, aiming at explaining organizational ethical or unethical behaviors and their origins (e.g., personality traits, biases, peer pressure, and organizational culture and practices; Prentice, 2014; Treviño et al., 2006). These two approaches to organizational ethics education often fall short of immersing students in concrete, applied learning experiences that mirror the complex, ambiguous, and context-dependent nature of the ethical dilemmas they are likely to encounter as future managers. Recent discussions in organizational ethics education have made the call for a more integrated pedagogy that bridges normative and behavioral approaches. In their conversation paper about business ethics education, De Los Reyes et al. (2017) discuss different approaches to realize this integration and propose the “spaghetti model,” which illustrates the inherent messiness of the distinction between normative and behavioral aspects of ethics, that are in fact inextricably intertwined like spaghetti strands and impossible to disentangle in practice. They situate this model within the virtue ethics tradition, emphasizing the cultivation of students’ holistic character development. However, the model remains largely cognitive in orientation, focusing on deepening students’ understanding of ethical principles and behaviors rather than fostering their active engagement with ethical situations. Its messiness, while conceptually rich, also represents a pedagogical challenge for educators seeking to translate it into practice.

### For a Pragmatist and Humanistic Approach to Organizational Ethics Education

We argue that moving beyond the dichotomy of normative and behavioral approaches to better embrace the complexity of ethics learning can be achieved through a pragmatist approach to organizational ethics education. Such an approach supports the humanization of business by engaging students in immersive activities and experiences that embody a “living ethics” stance (Racine et al., 2024; Solberg et al., 1995). Pragmatism in organizational ethics prioritizes the practical consequences of ethical decisions and actions (Pouryousefi & Freeman, 2021). As such, this intellectual stand recognizes that ethical situations in organizations are often complex and context-dependent (Moosmayer et al., 2019). Consequently, because ethical decision-making in organization is not a straightforward process, pragmatist thinkers acknowledge the value of learning from experience to continuously improve ethical practices (Singer, 2010). Therefore, training future managers in experiential settings supports them in learning how to consider stakeholders’ needs as well as how to navigate the unique, complex features of each ethical dilemma.

We also support a humanistic approach to ethics education, highlighting the human dimension of business and organizations (de Colle et al., 2024; Dion et al., 2022). In this perspective, organizations and businesses are seen as vehicles for human cooperation, where individuals come together, collaborate, and form communities (Melé, 2012). It stresses the importance of interconnectedness between individuals and the impact of interpersonal relationships on ethical considerations in organizational settings (de Colle et al., 2024; Dion et al., 2022; Giacalone & Promislo, 2013; Pirson, 2020). Ultimately, organizational ethics revolves around “the human desire to create value with and for others” (de Colle et al., 2024, p. 555). Therefore, rather than solely adhering to normative principles, organizational ethics education should prioritize ethical reflection and behaviors guided by empathy, care, and mutual respect (Cohen, 2012).

In line with the pragmatist philosophy of education (Dewey, 1997), and inspired by a humanistic view of ethics (de Colle et al., 2024), SpaceJam proposes a means to holistically develop students’ character via the cultivation of empathy and ethical reflection to support further ethical commitment and action beyond the classroom. It proposes a shift from learning to living or experiencing ethics to propel students into their managerial roles, equipping them with essential skills to confront the grand challenges of our time (Mailhot & Lachapelle, 2022).

## Fostering Empathy for Organizational Ethics Education

Research in psychology, social neuroscience, and biology converge to define empathy as an individual natural ability to share and understand the emotions of others (Decety, 2015). Scholars identify three dimensions of empathy: affective, cognitive, and motivational (Decety, 2015; Decety & Jackson, 2004; Leiberg & Anders, 2006; Martineau et al., 2020). The affective dimension of empathy is a spontaneous emotional experience of the emotions of others (Batson, 2009), while the cognitive dimension refers to a deliberate intellectual effort to put yourself in the shoes of other people and understanding their inner state (Decety & Yoder, 2016). The motivational facet of empathy, or empathic concern, refers to feelings of sympathy or compassion for others, that drive individuals to offer help or care to others (Batson, 2023; Eisenberg & Eggum, 2009), and is closely linked to engagement in pro-social behavior (Batson, 2012; Decety & Cowell, 2014). Although these dimensions are distinct and can be studied independently, they are deeply interconnected and mutually influence one another, collectively constituting the holistic human experience of empathy (Martineau et al., 2020).

Interdisciplinary research widely acknowledges empathy’s central role in morality and its positive impact on encouraging pro-social behaviors and inhibiting unethical conduct (Batson, 2012; Decety & Cowell, 2015). In an organizational context, empathy enables managers to consider the emotional ramifications of organizational realities, to understand the position of other stakeholders, and to signal ethical dilemmas, so as to better inform ethical decision-making processes (Jian, 2022; Martineau et al., 2020). Furthermore, empathy serves as both a motivation for justice and an inhibitor for aggressions and unethical behaviors by establishing a bedrock for care-based morality (Decety & Cowell, 2015; Decety & Yoder, 2016). Certainly, empathy presents ethical boundaries that can manifest through the introduction of biases and favoritism (Batson, 2012; Batson et al., 1995), or by excessively prioritizing others’ emotions, potentially resulting in moral fatigue among managers (Bolino & Grant, 2016). However, considering the unique challenges, circumstances, and culture of business schools, the importance of fostering empathy remains significant for organizational ethics education.

Indeed, empathy has emerged as a vital competency for future business leaders, increasingly recognized as essential within managerial education (Holt et al., 2017). For instance D. F. Baker (2017) highlights empathy’s role in shaping ethical decision-making processes among business students, suggesting that empathy directly influences managerial judgment, enhances moral reasoning, and equips students to navigate complex ethical dilemmas in their professional lives. Bertella (2025) broadens this perspective by connecting empathy explicitly to improved sustainability education. Finally, Zhou (2022) provides a critical review that emphasizes empathy’s educational value, particularly its ability to enrich learning environments, enhance interpersonal connections, and foster a deeper understanding of diverse perspectives. He suggests incorporating empathy systematically across educational programs, including business education, to support holistic professional and personal development.

Some initiatives geared toward teaching empathy in business schools have emerged in the past years (D. F. Baker, 2017). However, these initiatives are predominantly focused on the development of one dimension of empathy, either affective (Jeong et al., 2020) or cognitive (Berti et al., 2021). Yet, as suggested by Brown et al. (2010), business schools should give students diverse opportunities to develop ethical skills across their learning journey and thus include empathy development in their curricula.

## Introducing the SpaceJam Pedagogical Strategy for Organizational Ethics Education

In response to these calls, we have developed SpaceJam, a pedagogical strategy focused on nurturing empathy, promoting students’ ethical reflection, and cultivating ethical behavior. Its aim is to provide undergraduate business students with diverse learning experiences that deepen their understanding and application of organizational ethics across contexts. Here, we explore the underpinnings of SpaceJam, rooted in a pragmatist and humanistic view of ethics and ethics education, and outline its key pedagogical principles. As we show, the pragmatist perspective informs the structure and learning contexts of the strategy, while the humanistic view of ethics informs its contents and objectives.

### Contextualizing Ethics, Multiplying Learning Spaces

SpaceJam champions a pragmatist, dynamic approach to organizational ethics education, valuing experiential learning as a means to continually refine ethical practices (Dewey, 1997) and thus prioritizes the practical and contextual implications of ethical decisions and actions (Mailhot & Lachapelle, 2022; Pouryousefi & Freeman, 2021; Singer, 2010). Rather than adhering rigidly to theoretical frameworks and normative principles, it promotes adaptability to different contexts and integration of plural ethical values and practices. This approach equips future managers with the skills to navigate the complexities of ethical dilemmas through immersive engagement with real-world scenarios (Visser, 2019). In doing so, our integrative stance not only transcends both traditional approaches to organizational ethics education, either focused on normative principles or behavioral aspects of ethical phenomena (De Los Reyes et al., 2017), but also engages students in a range of experiential learning opportunities (D. A. Kolb, 2014), including dialogue (Shor & Freire, 1987), service-learning (Bringle & Hatcher, 1996; Godfrey et al., 2005), artistic expression (Rusu, 2017), and pedagogic catharsis (Schaper, 1968) as ongoing opportunities for self-reflection. These immersive pathways aim to engage students in moral quandaries, prompting them to critically examine their reactions and problem-solving approaches.

Consistent with this perspective, SpaceJam situates students’ learning within varied spaces, fostering an understanding that ethical issues in organizations and society are multifaceted and contingent upon specific circumstances, stakeholders, and resources (Moosmayer et al., 2019). In this regard, a stream of literature in pedagogy addresses and redefines learning spaces in the light of experiential pedagogy. A. Y. Kolb and Kolb (2005) introduce the notion of learning spaces as institutionalized social contexts in which learners move toward becoming part of a community. Various learning spaces exhibit differences in both social dynamics and material characteristics (Cayouette-Remblière et al., 2019; Holman et al., 1997). In our strategy, these spaces include the classroom, the home, and the community—each providing distinct yet interconnected contexts for ethical reflection and empathy development, consistent with Kolb and Kolb’s notion of learning communities. Each space is uniquely characterized by its physical attributes, sensory elements, and material arrangements and resources, offering distinct opportunities for learning, social interaction, and artistic engagement. Situating students in different learning spaces not only broadens their learning opportunities and experiences, but it also exposes students to a variety of realities, while giving them opportunities to develop a plural understanding and appreciation of the ethical issues affecting everyday life, organizations, and workplaces.

### Fostering Empathy Through Art-Based Methods

Building upon a pragmatist and humanistic foundation, our strategy emphasizes the cultivation of empathy and ethical reflection through art-based methods (Katz-Buonincontro, 2015; Nussbaum, 2010; Starkey & Tempest, 2009). Indeed, research from various fields suggests that empathy can be taught and learned (Feshbach & Feshbach, 2009). A large body of literature suggests that arts-based methods, including music, movies, literary works, and theater, can contribute to management education (J. A. Morris et al., 2005; Taylor & Ladkin, 2009; van der Hoorn & Donovan, 2023). More specifically, literature from different disciplines supports that exposure to arts can stimulate empathic dispositions, facilitating the development of imaginative, emotional, and aesthetic abilities (Decety & Cowell, 2014; Kidd & Castano, 2013; Nussbaum, 2010). In this regard, research indicates that employing artistic methods in education aids in the development of soft skills (Robbie & Warren, 2021), and system thinking (Molderez & Ceulemans, 2018). It is also noted to support students in consolidating learning (Springborg & Ladkin, 2018), addressing sensitive issues (Crowley et al., 2020), developing sensitivity to others, and providing an avenue for self-expression (Çetin & Güneş, 2021). Arts-based approaches have been successfully used to teach organizational ethics courses, mobilizing literature and novels (Michaelson, 2016; von Weltzien Hoivik, 2009), films and movies (Champoux, 2006; Skorin-Kapov & Benson, 2018), as well as theater (De Colle et al., 2017; Freeman et al., 2015), as notable mediums.

While numerous studies highlight the pedagogical value of the arts in fostering empathy and ethical reflection, research also points to several challenges associated with arts-based pedagogies. These include student discomfort with unfamiliar expressive modes, the need for supportive learning environments, and the difficulty of assessing subjective and interpretive outcomes (Freeman et al., 2015; Stavraki & Anninou, 2022). Such approaches also require educators to balance analytical and creative learning and, at times, to collaborate with arts professionals or institutions for effective facilitation. SpaceJam responds to these challenges by emphasizing ethical reflection over artistic technique, offering flexible modes of expression, and creating experiential learning contexts that integrate both cognitive and affective engagement.

### An Integrative Strategy for Actionizing and Humanizing Ethics Education

**Figure 1.**
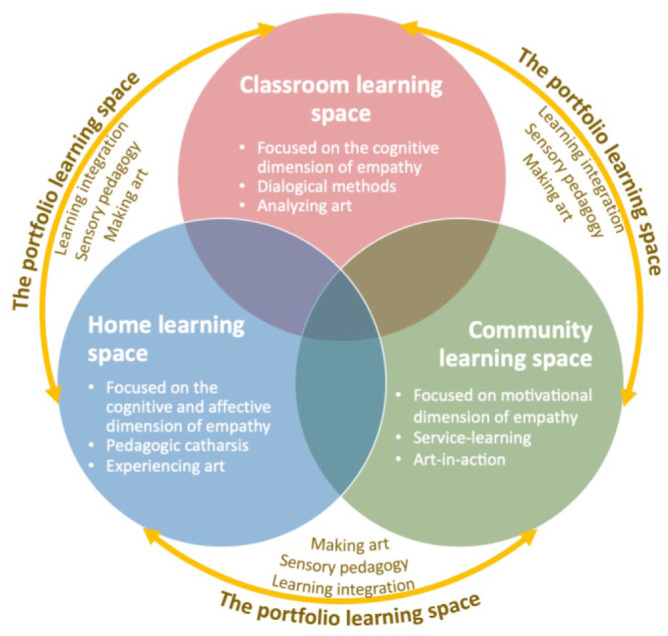
The SpaceJam integrative pedagogical strategy.

SpaceJam is an integrative pedagogical strategy that intersects these currents of thought and pedagogical methods. The strategy is illustrated in Figure 1. Following this approach, students are immersed into three different learning spaces—the classroom, their home, and the community—where they engage in different learning experiences and art-based pedagogical methods. Each of these spaces offers diverse learning opportunities primarily focused (but not exclusively) on nurturing a specific dimension of empathy fostered by specific pedagogical approaches and ways to engage with the arts that align with the unique material and social characteristics of these learning spaces (see Table 1 for each learning space’s specificity within the SpaceJam strategy).

**Table 1.** SpaceJam—Pedagogical Approaches and Outcomes.

SpaceJam	Classroom	Home	Community	Portfolio
Aim at	Sharing and listening	Feeling and pondering	Committing and engaging	Expressing and anchoring oneself
Material and social characteristics of the learning space	Promotes learning via the socialization with others (He, 2015; Johnson, 1982; Soltani, 2018) in a formal learning environment.	Serves as a private retreat, typically associated with familiarity and intimacy, which facilitates a dynamic between comfort and discomfort promoted, in most cases, by a sense of control and freedom over emotions and experiences (Abbott-Chapman & Robertson, 2001; Conradson, 2003).	Presents a public arena where students confront real-life situations and emotions beyond their immediate experiences (Biesta, 2012), connect with others, and address societal issues, encouraging involvement in community initiatives (Cohen, 2012; Fougère et al., 2020)	A tangible sketchbook, which serves as both material and artistic anchor and a nexus for integration, offering students a space to express themselves and reflect on artworks, class discussions, service-learning experiences, cultural events, etc.
Primary learning focus	Cognitive dimension of empathy	Cognitive and affective dimensions of empathy	Motivational dimension of empathy	Ethical reflection
Pedagogical approaches	Dialogical (Shor & Freire, 1987) and conversational learning (A. C. Baker, 2004; Sims, 2004) that facilitate not only self-expression but also active listening and appreciation of peers’ perspectives (Mejia, 2022).	Pedagogic catharsis, which refers to the powerful effect of engaging with artworks that can elicit strong emotions and even a sense of shock (Ezcurra, 2012; Schaper, 1968), prompting students to navigate these emotions in a supportive environment, while simultaneously encouraging them to reflect on why they experience them (Ayikoru & Park, 2019; Kearney et al., 2013; J. Morris, 2005).	Service-learning pedagogy (Godfrey et al., 2005) exposes students to different realities, fostering empathy and cooperative behaviors while supporting ethics education (Cohen, 2012; Fleckenstein, 1997). We view service-learning as an “experience in which students participate in an organized service activity that meets identified community needs and reflect on that service activity in such a way as to gain further understanding of course content, a broader appreciation of the discipline, and an enhanced sense of civic responsibility” (Bringle & Hatcher, p. 222).	Artistic expression (Rusu, 2017), crafting, and hands-on sensory pedagogy (Grittner, 2021; Vansieleghem, 2021) that enhance memory encoding and retrieval, benefiting students with diverse learning styles (Reiner, 2009; Wheeler et al., 2000).
Engagement with the arts	A forum for discussing artworks, exchanging viewpoints, mobilizing theoretical concepts, and discussing ethical dilemmas.	Exposure to diverse artistic works, ranging from novels to documentaries, students can explore ethical scenarios beyond their daily encounters (Crowley et al., 2020).	Museum or theater visits, offering firsthand encounters with artistic expression and immersive learning. Participation in an “art-in-action” project, creating a photo-reportage on working conditions and (un)happiness at work that encourages artistic engagement with their surroundings.	Engagement in multiple senses through artistic modes of expression for students to take ownership of their learning, anchoring new knowledge in self-reflection and crafting on topics that resonate with them. Various artistic mediums such as drawing, painting, collage, photography, poetry, or music enable students to express themselves while internalizing course content (Sousa, 2006).

The integrative core of the strategy is manifested through the development of an artistic portfolio, which serves as a fourth learning space in which students continually make sense of and reflect on their learning experiences across the other spaces^1^. Each week, students are required to complete two pages in their portfolio, following a structured format. First, on the left-hand page, they engage in a directed assignment, analyzing an artwork related to the class theme. Second, on the right-hand page, they tackle an open assignment, delving into a topic of their choosing loosely connected to organizational ethics. For the weekly open assignment, students are granted full autonomy and freedom to select both the topic they wish to explore and the angle of addressing it, as long as it fit within the confines of one page in their portfolio. In both assignments, students are encouraged to express themselves by using various artistic mediums such as painting, drawing, collages, poetry, or music (students have added links to playlists or recorded music to their portfolios). Once again, they enjoy full autonomy in selecting the artistic mediums that aligns with their preferences. Through the work realized in their portfolios, students crystallize learning by translating pedagogical content and experiences into their own words, art, and reality, thus consolidating empathic dispositions across learning spaces (Burke, 2006; Klein, 2018). Annex 1 presents examples of students’ work in the portfolio.

## Implementing SpaceJam

The pedagogical strategy was formulated and implemented within an elective undergraduate organizational ethics course at our university. The course was initially titled *Ethics and Management* and has since been renamed *Ethics in Action: Empathy, Organization, and Society* to better reflect its content and approach. This elective course is designed as a continuation of the mandatory *Ethics, Business Law, and Governance* course offered to all undergraduate business students. While the mandatory course relies heavily on lectures and case studies, the elective one puts students into immersive “living ethics” experiences through the SpaceJam strategy. Both authors of this article actively contributed to the conception, implementation, and teaching of the strategy.

Annex 2 summarizes how SpaceJam was implemented in this course for the Winter 2022 semester. The activities included into the course are presented for each of the classes and within each learning spaces presented before. The course’s artworks, themes, and activities are evolving from one semester to another, to adapt to the social environment.

The strategy aims not only at integrating the four learning spaces presented above, but also at navigating through these spaces. In Annex 3, we present an example of a sequence of learning activities used in the course. Navigating spaces through this sequence of activities allows students to have a more complete and embodied understanding of the ethical issues covered in class.

## Impacts on Learning

SpaceJam was implemented, and documented over a span of 3 years, from January 2022 to December 2024, over three cohorts of students in our university. All students had previously completed a mandatory course in ethics in which they had learned about the major ethical theories using a more traditional pedagogy based on lectures and case studies. The course attracted a diverse cohort predominantly consisting of students majoring in management and sustainable development, although there was representation from fields such as finance, accounting, marketing, and more. Most of them were senior students and enrolled in this class in order to deepen their learning in ethics, using a different experiential approach.

The population of students in the bachelor’s degree program consists of 80% Canadian citizens (comprising 78% citizens and 2% permanent residents), along with 20% international students hailing from various countries worldwide, notably with a significant representation from Europe and North Africa. Data from 2022 indicates that 47% of students in the bachelor program were women, 53% were men.

### Documenting the Impacts of the SpaceJam Strategy

We gathered three types of evidence to document the impact and effectiveness of our pedagogical strategy on students’ learning and engagement. This evidence consists of excerpts from the course teaching evaluations, of contents from students’ portfolios, and of verbatims transcribed from semi-structured interviews conducted with students as part of our research project. All participants to this project were students enrolled in the elective organizational ethics course during the Winter 2022, Fall 2022, and Fall 2023 semesters, in the business administration bachelor program at a large business school in Canada.

This research was approved by the Research Ethics Board of our university, and we obtained written informed consent from all participants (interviews and portfolio sharing). Each dataset tracks student testimonials of their learning experience at different points in time and under differing conditions. If teaching evaluations provide on-the-spot, anonymous reflections at the very end of the semester, portfolios offer ongoing evidence into learning activities and reflections throughout semesters, while interviews capture retrospective and more in-depth reflections on students’ learning experiences. Multiplying sets of data recorded at different points in time and under different conditions offers a range of alternative viewpoints, which, ultimately, allow data triangulation.

First, we were able to obtain qualitative evidence on the learning impacts of our new pedagogical strategy from the course teaching evaluations, which have been completed by 30 respondents out of a population of 42 students enrolled in the course of the Winter 2022 semester (71% response rate), by 11 of the 20 students in the Fall 2022 semester (55% response rate), and by 19 of the 35 students in the Fall 2023 semester (54% response rate). Teaching evaluations provide testimonies on learning experiences at the very end of each semester, and the anonymous nature of evaluations allows for nuanced reflections on the appreciation of the pedagogical strategy.

Second, we received permission from 50 participants across the three cohorts to use the content of their portfolio for our research project. We gained access to 25 portfolios of the first cohort (60% response rate), of 13 from the second cohort (65% response rate), and of 12 of the third cohort (34% response rate). The pages of all 50 portfolios were digitalized to enable analysis. The content of the portfolios allowed us to gather evidence about the learning activities and reflections carried out by the students during the semesters.

Third, we conducted a total of 37 semi-directed interviews across the three cohorts of participants. 16 interviews were conducted with students from the first cohort (39% response rate), 9 interviews were conducted with the students from the second cohort (45% response rate), and 12 interviews from the third cohort (34% response rate). In average, interviews lasted 75 min. Interviews were conducted post-semester by an impartial, external research assistant, without any prior pedagogical relationship with the participants, in accordance with the guidelines of our institution’s Research Ethics Board. This promoted evidence-gathering that minimized any possible bias and power-play between teachers/researchers and students/participants (Blenker et al., 2014). Interviews were recorded and transcribed verbatim to facilitate analysis. The interviews focused on the students’ retrospective thinking on their learning experiences and sought to capture their impressions of the pedagogical approach and activities mobilized in each space, their views on the purpose of those experiences, and their overall assessment of the learning they made in the course.

### Data Analysis

Deductive qualitative data analysis (Miles et al., 2014) provided a structured and systematic approach to assess the effectiveness of the pedagogical strategy from a student-centered perspective. We conducted thematic analysis (Fereday & Muir-Cochrane, 2006) of the teaching evaluations, the portfolios, and the transcribed interviews. We categorized participants testimony into themes based on the theoretical foundations and learning spaces of the pedagogical strategy. Our coding protocol focused on the examination of students’ perception regarding the learning outcomes attained within each specific space and across spaces, with a specific focus on the different dimensions of empathy (cognitive, affective, and motivational). Table 2 below presents our general thematic coding grid and process.

**Table 2.** Coding Grid and Process.

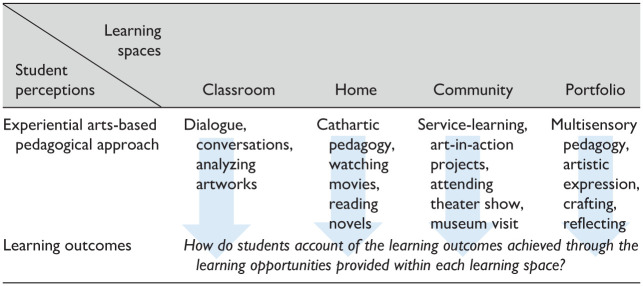

We used the MAXQDA software for coding data. A member of our research team first coded the interview transcripts using the coding grid derived from the theoretical foundations of the *s*trategy. The interview verbatims were subsequently double coded by a second researcher to ensure reliability. Discrepancies were discussed and resolved during research meetings. Results obtained from the analysis of the interviews transcripts were further corroborated by thematic analysis of the teaching evaluations and the portfolios. Triangulation across these three sets of data helped us compare data from multiple sources and perspectives and cross-validate findings to enhance this study’s credibility and to ensure a comprehensive understanding of the students’ experiences of the pedagogical strategy.

In the following section, we present qualitative evidence of the effectiveness and impacts of SpaceJam on students’ experience and learning. We report on the results of our analyses for each learning space, focusing on the pedagogical approach, the use of the arts and their impact on student learning. This section culminates with the presentation of a comprehensive learning process model that illustrates how anchoring learning gained through the strategy promotes the expansion of empathic dispositions and ethical behavior in students across contexts.

### The Classroom Learning Space

Overall, in the classroom learning space, the students indicated that the dialogical approach used in the classroom learning space helped them develop sensitivity to different perspectives and points of view. This awareness of others and of diversity provided them with valuable abilities for navigating complex contemporary business issues, which they will have to face in their future work as managers. More specifically, class discussions enabled students to develop stronger social abilities, both in terms of listening to others and of sharing their opinions and points of view. Students reported that through class discussions, they were given opportunities to learn how to listen and consider divergent points of view, to understand the rationale and emotional state behind their colleagues’ positions, and to reflect on their own standpoint regarding the issue in question. Finally, the pedagogical approach and the use of arts in the classroom learning space supported the development of empathic dispositions in students. This approach engaged students in a process that encouraged them to refine and expand their understanding of diverse situations and to consider others’ perspectives without judgment. Table 3 presents verbatims corroborating these results.

**Table 3.** Students’ Perceptions of the Classroom Learning Space.

Approach and outcomes	Students’ perceptions
Conversational learning	“[as a business school student], I can see that it’s important to have a course like this. If you want to work in sustainable development, you have to understand other people’s differences, because everyone has a different point of view. You know, there are still climate skeptics out there, and we’re going to face challenges in this area in our work as managers in the business world.” (Interview with participant #13, cohort 1) “[. . .] everyone had different experiences of, either working, or coming from Europe, so we were exposed to a lot of different points of view, so it was good to be exposed to different opinions and to accept a bit how everyone thinks.” (Interview with participant #4, cohort 1)
Specific learning outcomes: Ability to listen and understand others	“I really think [that the course develops] the ability to listen to other people’s points of view without judging them, even without saying it out loud, but really listening to what the other person has to say without always trying to contradict, to find the opposite of what they’ve just said.” (Interview with participant #15, cohort 1) “I really like this approach, which is very different from what you find in other courses. [. . .] It was really more advanced, really focused on reflection and precisely on a process of active listening, where we try to take a little more interest in what people are saying, accept other people’s different points of view, try to give our own, and then analyze.” (Interview with participant #19, cohort 2).

### The Home Learning Space

In the home learning space, the students have reported that watching films and documentaries, as well as reading a play, has given them access to realities they don’t face or experience on a daily basis. As presented in Table 4, they mentioned that the cinematic medium provided them with a better means of imagining the realities being depicted, as well as of projecting onto others’ lives and identifying with the characters. Students also recounted that these artworks triggered shock and awe, while for others they provoked tears and a sensation of helplessness. In line with pedagogic catharsis, the students mentioned that the emotions felt as a result of watching films triggered empathy for the characters portrayed in the artworks and left a lasting imprint on them. In some cases, these emotions and recognition of others’ reality initiated an in-depth reflection process, which they then shared with members of their inner circle.

**Table 4.** Students’ Perceptions of the Home Learning Space.

Approach and outcomes	Students’ perceptions
Pedagogic catharsis	“You know, [about the documentary] *Wasteland*, well I don’t really live in a poverty-stricken slum. And Erin [Brockovich], she’s a single mother struggling to make ends meet and all, you know I don’t think I can really put myself in their shoes in my daily life. [. . .] But we’re able to imagine them when it’s represented in a film, it’s like easier to live it through the character, I think.” (Interview with participant #8, cohort 1) “About the poverty depicted in the *Wasteland* documentary] “It’s something you don’t experience on a daily basis. I mean, when you live in Montreal, you don’t live [in this poverty], but to see it [in a documentary], to see people who live it, to see the life they lead, I think, it develops empathy because you put yourself in the place of others, and then you try to understand the emotions they live and the difficulty of their life.“ (Interview with participant #21, cohort 2) “I’m talking about the movies we had to watch. Many of them plunged us deeply into the emotions of the characters. For example, in the documentary *Wasteland*, we really understood what they were going through.” (Interview with participant #32, cohort 3)
Specific learning outcomes: Ability to feel others’ emotions and imagine their reality	“Personally, I’m a big fan of documentaries because I think they’re more emotional than films. And so the documentaries we had to watch, particularly the one on *Wasteland* in Brazil, was something that really moved me, I wouldn’t say to tears, but it really disturbed me [. . .] I think I really thought about it, I really had it in my head for a long time.” (Interview with participant #15, cohort 1) “There are some artworks that brought tears to my eyes. Others I had [. . .] a lot of empathy for the character. And I put myself in his shoes. It made me question myself and have dialogues within my own family about the work, and I even watched it with other members of my family.” (Interview with participant #6, cohort 1)

### The Community Learning Space

In the community learning space, students noted that helping others through service-learning took them out of their regular routine and gave them a sense of accomplishment. They learned that helping others was just as rewarding for those who benefited from it as it was for themselves, since they gained a sense of pride and joy from the experience. Service-learning helped students discover new horizons, while simultaneously opening their minds and undoing stereotypical assumptions about some social groups (e.g., the elderly, the homeless, people with disabilities, etc.). It also enabled students to see how people with different backgrounds can work toward common objectives, even if they don’t follow the same paths. Our analyses revealed that engaging students in an art-in-action project (i.e., a photo-reportage on workers in their community) promotes lessons similar to those associated with service-learning. Finally, our analysis revealed that encouraging students to take action, either through service-learning or an art-in-action project, influences students to adopt helping or caring behaviors. Students reported that the activities proposed in the community learning space helped stimulate accountability to others and push them to adopt a solution-oriented mindset that will be useful in their future work. We present verbatims illustrating these results in Table 5.

**Table 5.** Students’ Perceptions of the Community Learning Space.

Approach and outcomes	Students’ perceptions
Service-learning	“I not only knew that I was a great help to the ladies, but a sense of pride followed me the entire time. On a more personal level, it felt good to do something for others. Telling people around me that I was volunteering made me feel proud, because it was a selfless act, but more importantly, it was something that stood out from a young adult’s everyday life.” (Excerpt from portfolio #27, cohort 1) “In a way, I’ve learned that I can make a difference for someone [. . .] that a small action can really bring happiness to someone. And that it’s also good to get out of your comfort zone and do things like that, and get out of my house. Because last Winter, I could have stayed home 24 hours a day. Especially with the Winter, the pandemic, we weren’t doing anything. I think it got me out of my house and helped people at the same time. . . it was as if it helped two people.” (Interview with participant #8, cohort 1) “I was coming out of that moment, then I felt fulfilled, it made me feel indescribably good.” (Interview with participant #36, cohort 3)
Specific learning outcomes: Helping or caring behaviors	“[The service-learning project] has also given me a better understanding of accountability. Every week you realize that you are calling someone who is vulnerable and fragile. During the calls, you have to listen, and you cannot judge the person for their actions. Since their reality is already difficult, you must be solution-oriented and see how you can help them have a better day and brighter moments.” (Excerpt from portfolio #4, cohort 1) “What I will remember most of all from this experience is the kindness of the people around us. We sometimes have reservations or quick judgments about the people we meet. Whether it is age, nationality, or economic situation, we are all biased in some way. I feel like I’ve pushed back against those stereotypes and noticed that we are all human . . . ” (Excerpt from portfolio #11, cohort 1) "I decided to volunteer with the elderly in a nursing home. And it’s true that I’ve been in a student environment for several years now, and in general it’s the kind of people I’ve been around a lot on a daily basis. As a result, I may have had less empathy for them [the elderly], for what they were going through. But then, just by interacting with them, I realized what their daily lives were like, and just how difficult it was for so many people. And even now, [. . .] when I see elderly people, [. . .] it touches me immediately more than before” (Interview with participant #30, cohort 3).

### The Portfolio Learning Space

While the results presented for each space align with the literature, the main contribution of this paper lies in the results obtained regarding the artistic portfolio. Students reported this portfolio to be the integrative core and anchor throughout their learning journey. They noted that this unique and original learning device took them out of the academic realm to which they had been accustomed to in a business school. They noted that the portfolio presented an opportunity to develop their creativity and reflection in a less restricted way. This encouraged them to put more effort and time into this project, which was not so much a straightforward assignment as a free, personal, and meaningful learning opportunity. Our analyses revealed that the portfolio was used by students as a means of taking ownership of their learning. Participants saw it as an opportunity to draw parallels with their interests, experiences, and personal lives. They also perceived it as a way of cultivating moral values.

In addition to providing a means of tailoring the learning process to each student needs and sensibilities, participants revealed that the portfolio was instrumental in the development of ethical reflection as well as in the retention of valuable new knowledge and skills. In line with the premises of sensory pedagogy, students explained this effect as a result of the time they spent creating their portfolio, the hands-on nature of the exercise, and the anchoring of their reflections in a material and concrete endeavor situated in lived experiences. Students also explained this enhanced memory and retention of information as arising from the material and creative process intrinsic to the crafting of their portfolio. In this way, the creative process through which they transposed ideas, reflections, or emotions into images or artworks gave them the opportunities for imaginative exploration. This helped students to connect what they had learned to a strong mode of expression, a powerful image, or an inspirational artistic gesture, thereby materializing their learning and anchoring it in memory, which increased retention. Table 6 presents illustrative verbatims regarding the portfolio.

**Table 6.** Students’ Perceptions of the Portfolio Integrative Learning Space.

Approach and outcomes	Students’ perceptions
Hands-on sensory pedagogy/crafting	“ [. . .] it allowed me to get out of a theoretical framework where we’re used to having an analysis with a certain structure and using the same tricks we use in all our analyses to make it look good. [. . .] The fact that it was set up as a [free assignment] you make a portfolio, it’s art, you do a bit of what you want, I found that it broke down that structure and left room for more creativity. I found that very good. I find that I’ve written things that are much deeper there than what I would have put into a traditional management analysis assignment, let’s say.” (Interview with participant #14, cohort 1) “I’m pretty sure I spent a lot more time, even though it wasn’t required maybe, I spent a lot more time on this course than my other courses, but almost with pleasure. I loved doing it, whereas the other ways of learning, I’m not like excited about doing my five-page essay. It’s a bit boring, you do it and then you forget about it. Whereas maybe because it’s more manual [. . .] maybe it sticks in your brain more too.” (Interview with participant #17, cohort 2)
Specific learning outcomes: Reflection, and learning integration and retention	“It forced me to think deeply . . . I really pushed myself to do this portfolio and I think I did it more for myself than just for the course. Because [. . .], it really took me out of the school reality, so I took the opportunity to do it for myself and not just as a task like math . . . And also, it allowed me to have discussions with people around me.” (Interview with participant #7, cohort 1) “It helped me to concentrate a little, to refocus, to let my creativity speak for itself, my creativity and what’s in my head, that, I don’t know, it appeased me. [. . .] I think that for certain types of students, it’s very, very cool [. . .] I’ve been diagnosed as ADHD and hypersensitivity, so it helped me gather my ideas around a common thread. (Interview with participant #26, cohort 3) “When you put an effort like I put into my portfolio to make art, then paint, then put time into it, it makes it so that I’m going to remember much longer the movie I watched to make that, because I put time into it, then I had to make connections with other things to be able to find things that inspired me for the drawing I wanted to make or the painting I wanted to make. I find it’s a good way to really anchor that in your brain.” (Interview with participant #20, cohort 2) “And if you push it a little bit in terms of memory or whatever, I think that in the learning process, I retained more information by doing this kind of creative work that you can find in the portfolio than if I had just taken notes and then revised for an exam or whatever. In the sense that since there was this creative process where I had to use my imagination, then mix a bit different ideas, different creations at the same time, it helped me retain a lot more theories. [. . .] I’ve got a friend who’s doing the core course in ethics, and I was able to help him with his course without even having to go back into the material, just because I remembered the ethical theories, how to apply them, which theorist, what his position was in relation to that.” (Interview with participant #19, cohort 2) “I find that everything I learned and put down on paper, and then made a concrete effort to explain it [artistically], just stayed in my brain. I think that’s a great thing about this course, because everything we did stay in my brain because it was concrete.” (Interview with participant #21, cohort 2)

## Discussion

### SpaceJam: Anchoring and Expanding Ethics Learning

**Figure 2.**
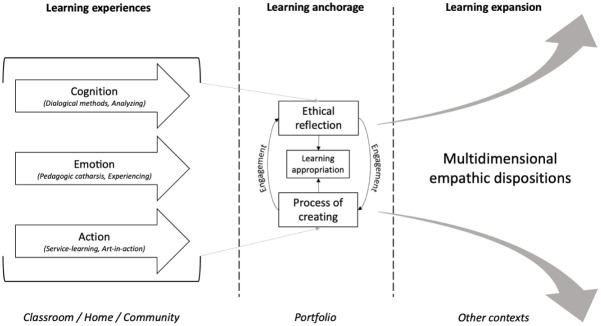
SpaceJam as a process of learning expansion.

Our findings highlight the importance of multiplying learning spaces, approaches, and opportunities for anchoring and expanding ethical skills and behaviors, such as empathy, ethical reflection, and judgment. As illustrated in Figure 2, the SpaceJam strategy engages students in a multidimensional learning process that is first rooted in immersive, interpersonal, material, and artistic experiences within several learning spaces. Each space features learning experiences that mainly focus on a specific learning dimension, be it cognition, emotion, or action. Our findings demonstrates that the artistic portfolio provides an important material anchor for catalyzing the learning generated within these experiential activities. Our analysis suggests that artistic expression, which entails both handcrafting and the process of creating and reflecting, enables students to appropriate learning and anchor new knowledge and soft skills in a durable way. Finally, our study shows that this process of material anchoring facilitates the subsequent transfer and expression of these newly acquired skills across other contexts.

### Transferability of the pedagogical innovation

We believe the SpaceJam pedagogical strategy is transferable to other pedagogical contexts: in other business schools, in other courses, as well as in the context of continuing training programs in organizations. The key idea behind this strategy is to stimulate the development of plural soft skills by reimagining (Kersh, 2015) and integrating learning spaces, and by using artistic approaches that reflect the materiality of the different learning spaces. Although developed to foster empathy and ethical reflection, we can well imagine SpaceJam being mobilized in a leadership or organizational behavior course. Such a strategy could also be used to develop the soft skills of future consultants or entrepreneurs in courses offered at undergraduate or graduate levels (MSc or MBA).

In order to transfer the SpaceJam pedagogical strategy to other contexts, it is important to first identify the learning objectives, the soft skills to be developed, as well as the learning spaces to be explored. In our case, these spaces are the classroom, the home, and the community. In an entrepreneurship course, for instance, a teacher might want to mobilize other spaces such as students’ start-ups, incubators, the business community, etc. The key is to identify learning spaces that can promote different modes of learning and engagement: affective, cognitive, action, and self-expression. Based on the learning objectives and spaces identified, educators should design activities and pedagogical methods tailored to the unique social and material characteristics of each space, as well as develop sequences that enable effective navigation across these spaces. Finally, it is necessary to define how the portfolio of artistic self-expression will articulate the learning spaces and integrate the pedagogical activities to crystalize learnings and foster the development of new soft skills. The originality of SpaceJam lies in the coherent integration of different approaches and learnings made in different spaces, which are materialized in the portfolio.

### Troubleshooting SpaceJam

SpaceJam is both a dynamic and evolving innovation. Continuous monitoring and evaluation through diverse data gathering methods allowed for adjustments and improvements, ensuring our pedagogical strategy was meeting both the learning objectives of the elective course and students’ needs. During the implementation with the first cohort, we were called upon to modify two main elements of the strategy: (1) the workload, which was too heavy for many students, and (2) the evaluation criteria, which had to be clarified, especially for assessing artistic expression and creativity in the portfolio. Here are some excerpts from the student evaluation of the course from the first cohort to illustrate these two issues:

The readings and resources for each class are really interesting and help illustrate and preview the concepts covered in class. However, the workload is much higher than other courses and required me to dedicate considerable amount of time to applying myself and producing something that lived up to my expectations and the expectations of the course. (Excerpt from teaching evaluations, cohort #1)

I like the idea of the portfolio project. However, I find the workload to be quite high. We have to do several pieces of writing each week as well as a freestyle work of art. We have to do our required service-learning hours as well. Still, I really like the idea. (Excerpt from teaching evaluations, cohort #1)

The best course in my BAA. Very interesting conversations and the artworks were well-chosen. Lacks a bit of clarity in the criteria for evaluating the Scrapbook (but I think that could be easily clarified). (Excerpt from teaching evaluations, cohort #1)

I enjoyed the resources (movies, readings, documentaries) to do every week. The discussion-based classes were very enriching and interesting. I really felt that I was able to expand my learning. However, the evaluation criteria for the assessments could be more detailed and clearer. (Excerpt from teaching evaluations, cohort #1)

Regarding the first point, we opted to enhance flexibility for students throughout the semester by making two weekly assignments optional, thereby adjusting the workload. Regarding the second point, the majority of students from the initial cohort noted that they lacked experience engaging with artistic mediums—such as visiting museums or attending theater performances—prior to the course. In this line, they expressed skepticism regarding the grading criteria for the artistic component of the portfolio within the context of a business school. For the second cohort, we refined the grading criteria for portfolio assignments. Evaluation now emphasizes the relevance and depth of students’ analyses and reflections, their integration of theoretical and experiential learning, and their efforts in effectively conveying their ideas through artistic expression, rather than judging their artistic talent or the aesthetic quality of their work. To this end, we revised the grading criteria for artistic expression, allocating 15% of the grading to each portfolio assignment for this purpose. Therefore, the feedback and insights gleaned from the initial cohort, coupled with the continuous, dynamic reflection between research and practice, have been instrumental in refining the pedagogical strategy, as well as its theoretical and ethical underpinnings.

### Key Success Factors in Implementing SpaceJam

We have identified five critical elements to successfully implement our pedagogical strategy in a course: (1) a small to mid-size group; (2) teacher’s and students’ commitment; (3) clarity in evaluation criteria; (4) course content adaptability; and (5) teacher’s responsiveness.

#### A Small to Mid-Size Group

In order to effectively implement SpaceJam, the group size should ideally be limited. We found that a group size of 25-35 students is optimal to facilitate in-class discussions and to be able to keep track of each student’s progress and needs. Such a group size supports teachers in developing a richer pedagogical relationship with their students and in acting out of mutual empathy as they engage in a process of developing their students’ empathic dispositions. In a more pragmatic view, a small to mid-size group also results in more efficient management of the service-learning activities. It also eases the task of evaluating the portfolio.

#### Teacher’s and Students’ Commitment

As noted above, this pedagogical strategy requires a substantial commitment from both instructors and students. Because it mobilizes multiple experiential approaches, it demands considerable effort from the teacher to design meaningful learning activities while following up on the content and adapting it in response to the needs and interests of the group. Reciprocally, students’ commitment must also be high. The strategy requires a high level of personal involvement in each learning space with the aim of internalizing new soft skills. The students also have a steady workload throughout the semester, in addition to the hours devoted to service-learning activities.

#### Clarity of Evaluation Criteria

The pedagogical strategy of the course—particularly the portfolio assignments—poses an inherent challenge for instructors in terms of evaluation. The very nature of the artistic assignments requires an evaluation based on efforts and not on the final visual product. Moreover, the portfolio provides students with considerable freedom to select topics and artistic media of their choice, which can complicate the task of ensuring fair and consistent grading.

To address this challenge, evaluation modes and criteria are clearly defined and communicated at the beginning of the semester. Instructors emphasize that the portfolio assignments are not evaluated for artistic talent, as this is not an arts course. Instead, the focus of evaluation lies on the quality and relevance of students’ analyses and reflections. Artistic expression is then considered insofar as it demonstrates effort, originality, and meaningful connections between ethical concepts and artistic form.

To promote inclusivity, the artistic component of the SpaceJam strategy is deliberately designed to accommodate a wide range of student backgrounds and comfort levels. Because no formal artistic training or technical support is expected or required, inclusivity is achieved through the portfolio’s flexible structure, which allows students to select mediums that feel accessible and meaningful to them—from written reflections or collages to poetry, photography, or music. Each student can therefore engage at a level consistent with their interests and abilities. This flexibility functions as a built-in support mechanism, allowing all students to participate meaningfully regardless of prior artistic experience.

Evaluation emphasizes three main criteria: (1) the relevance and depth of students’ analyses and reflections, (2) the integration of theoretical and experiential learning, and (3) the effort and effectiveness with which ideas are conveyed through artistic expression (limited to 15% of the grade). These criteria are applied consistently across all course assignments and discussed with students at the beginning of the semester to ensure transparency and fairness. As an illustration, the grading rubric used for the weekly left- and right-page portfolio assignments is presented in Annex 4.

#### Course Content Adaptability

The SpaceJam pedagogical strategy requires an update on an annual basis so that the topics, artworks, and cultural activities presented and discussed are up-to-date and resonate with students. Pedagogical activities that are relevant to topicality and students’ priorities and sensitivities help to establish a learning community in the classroom and to engage students in their learning journey. In addition, by situating students in front of issues that affect them on a daily basis, we encourage them to further express themselves, to take a stand, and to engage in reflections on their self, their role in organizations, and in society at large.

#### Teacher’s Responsiveness

Finally, the dynamic and evolving nature of the pedagogical strategy requires a great deal of responsiveness on the part of the teacher. Since SpaceJam is based on the interaction between the students and their environments, it must propose activities that are relevant to students’ daily lives and to global and regional news. Teachers must therefore always be both attentive to the needs and interests of their students and be attuned to the headlines in the media, cases in the news about ethics, cultural activities being offered in the community, etc. Consequently, teachers using the SpaceJam strategy have to constantly adapt their classroom discussions and activities to reconcile these realities.

### Limitations and Future Developments

This study was conducted in a Canadian business school with a French-speaking and internationally diverse student population. While this context provides valuable insights into a multicultural learning environment, the findings remain embedded in a specific institutional and cultural setting where artistic and reflective pedagogies are still emerging as complementary approaches to management education. Implementing SpaceJam effectively also presupposes an educational environment that is open to creative or non-traditional teaching methods, which may not be the case in all business schools or higher education systems. Contextual factors such as class size, disciplinary traditions, and cultural norms regarding artistic expression may influence how the strategy is implemented and perceived. For instance, students in cultures with more hierarchical or examination-oriented learning traditions might engage differently with creative, open-ended tasks.

At the same time, one of the strengths of the SpaceJam strategy lies in its adaptability. The approach can be tailored to diverse educational environments by varying the learning spaces, artistic mediums, discussion topics and service-learning components to fit local pedagogical cultures and institutional realities. Such flexibility allows educators to preserve the strategy’s ethical and creative core while aligning it with the resources, values, and expectations of their specific contexts. Future research could therefore investigate how our pedagogical strategy might be adapted and transferred across institutional and cultural contexts—by adjusting artistic media, assessment processes, or facilitation methods—to ensure both cultural relevance and pedagogical effectiveness.

Finally, as this article focuses on presenting an comprehensive instructional design and its impact using short-term qualitative data, it does not assess the long-term impact of the pedagogical strategy on students’ ethical development and sustained ethical behavior. Examining such outcomes would require longitudinal or mixed-method research designs capable of tracking graduates beyond the classroom and across career stages—a scope that exceeds the objectives of the present study. Future research could build on our findings by exploring whether the empathy and ethical reflection fostered through SpaceJam endure over time and influence graduates’ professional judgment, leadership practices, and ethical behavior in organizational settings.

## Conclusion

This article introduces SpaceJam, an innovative pedagogical strategy that emphasizes the benefits of a pragmatist, humanistic, and integrative approach to organizational ethics education. By synergistically integrating diverse learning approaches across varied learning spaces, fostering experiential engagement with empathy in its multiple dimensions, and coupling ethical reflection with artistic self-expression, the SpaceJam strategy enhances meaningful ethics learning outcomes.

At a time when societies face increasingly complex global challenges, ethical education for future business leaders is indispensable. SpaceJam not only represents a novel pedagogical framework but also actively cultivates students’ capacities for empathy, ethical reflection, and critical thinking. Ultimately, this approach equips learners to transcend traditional boundaries, embrace diversity, and thoughtfully address the nuanced ethical issues that characterize contemporary organizational life.

## Figures and Tables

**Annex 1.**
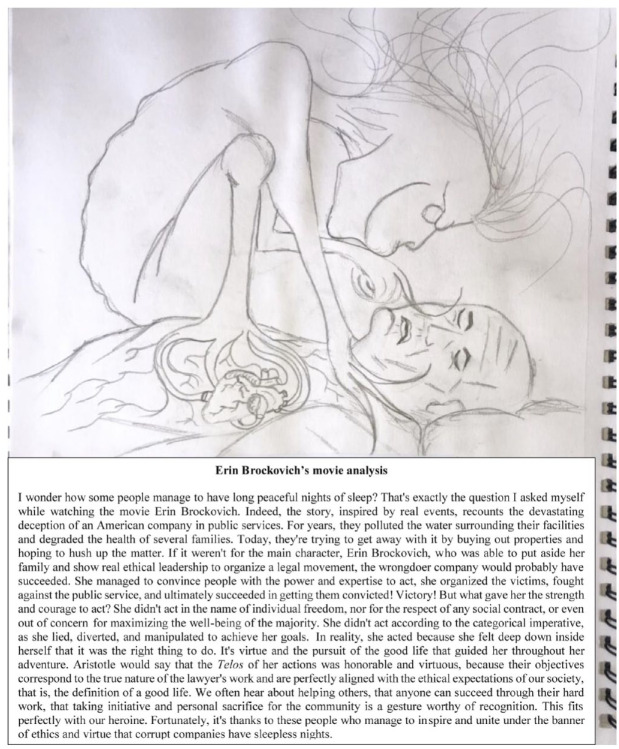
Examples of students’ work in the portfolio. (a) Analysis of a movie (portfolio #17, cohort 1).

**Figure:**
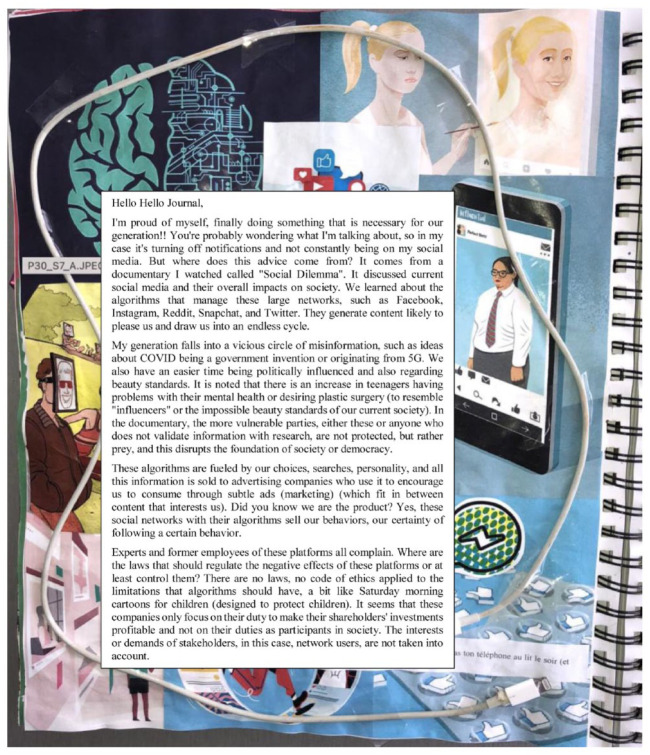
(b) Analysis of a documentary (portfolio #30, cohort 2).

**Figure:**
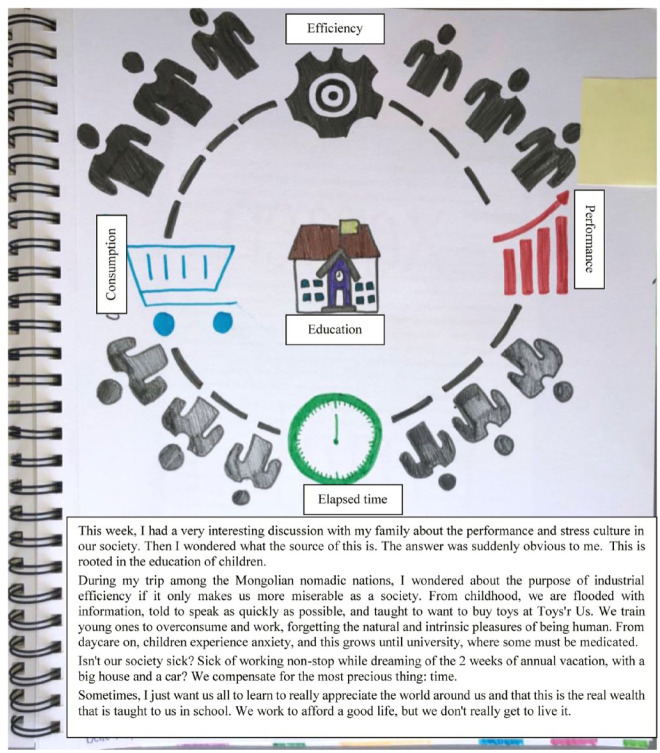
(c) Open assignment (portfolio #16, cohort 1).

**Figure:**
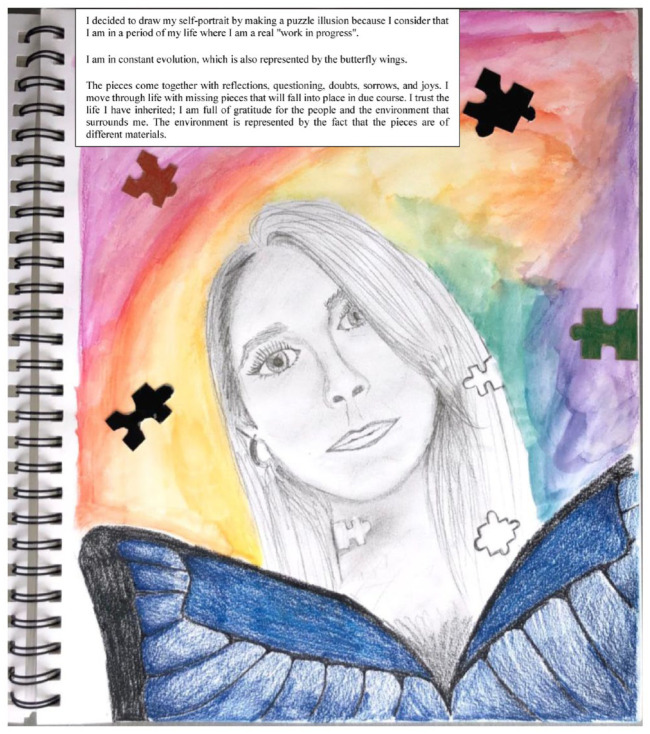
(d) Self-portrait (portfolio #12, cohort 1).

**Figure:**
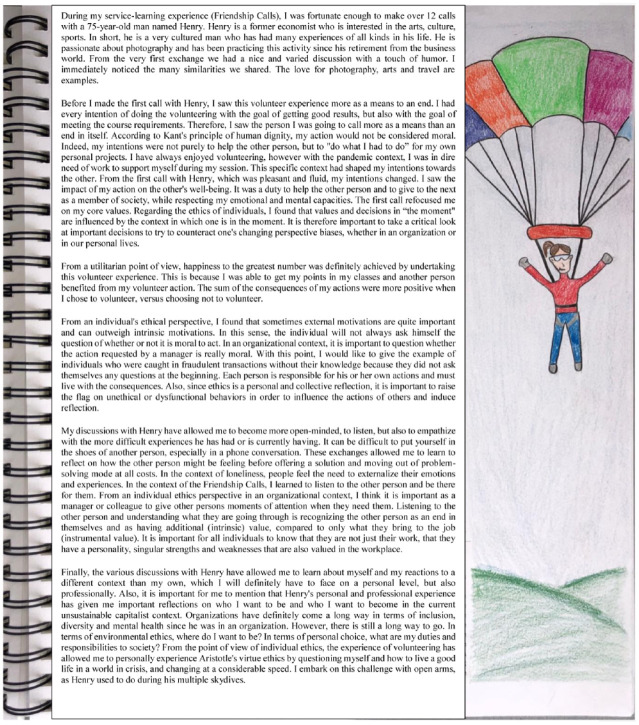
(e) Reflection on service-learning (portfolio #12, cohort 1).

**Annex 3.**
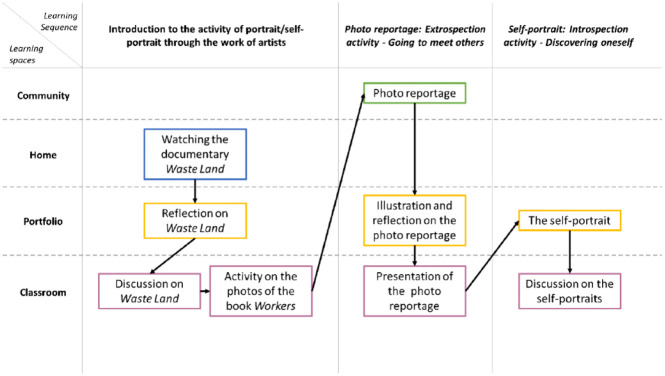
Navigating across learning spaces: an example of a sequence of activities.

## Appendix

**Annex 2.** The course “Ethics and Management” Winter 2022: Planning and activities.

Learning Spaces Classes	Community	Classroom	Home	Portfolio
BLOCK A: Business ethics				
*Class 1:* Introduction		• Watching an episode of *The Good Place* • Introduction to film analysis		
		• Introduction to the service-learning approach in the community		
*Class 2:* Organizational integrity	• Beginning of service-learning activities	• Discussion of the latest news • Oral presentation on the movie *Wall Street* • Discussion on integrity	• Watching the movie *Wall Street*	• Analysis and illustrations of the movie *Wall Street* • Open reflection on a topic of their choice
*Class 3:* Ethical climate and culture of organizations	• Service-learning activities	• Discussion of the latest news • Oral presentation on the movie *The firm* • Discussion on ethical culture in organizations	• Watching the movie *The Firm*	• Analysis and illustrations of the movie *The Firm* • Open reflection on a topic of their choice
BLOCK B: Empathy and contemporary management issues				
*Class 4:* Working conditions	• Service-learning activities	• Discussion of the latest news • Oral presentation on the documentary *Waste Land* • Analysis of photographs of workers around the world	• Watching the documentary *Waste Land*	• Analysis and illustrations of the documentary *Waste Land* • Open reflection on a topic of their choice
*Class 5:* Happiness at work	• Service-learning activities • Photo reportage on workers	• Discussion of the latest news • Oral presentation on the podcast on working conditions • Presentation of one photo from each student's photo reportage	• Listening to a podcast on working conditions	• Analysis and illustrations of the podcast on working conditions • Presentation of the photo reportage on the workers
*Class 6:* Empathy and emotional intelligence	• Service-learning activities	• Discussion of the latest news • Oral presentation on the documentary *Amazon* • Empathy and self-portrait exercise • Open dialogue exercise	• Watching the documentary *Amazon Empire: The Rise and Reign of Jeff Bezos*	• Analysis and illustrations of the documentary *Amazon Empire: The Rise and Reign of Jeff Bezos* • Self-portrait
*Class 7:* Future of work and technologies	• Service-learning activities	• Discussion of the latest news • Oral presentation on the documentary *The Social Dilemma* • Mindfulness Meditation Session	• Watching the documentary *The Social Dilemma*	• Analysis and illustrations of the documentary *The Social Dilemma* • Open reflection on a topic of their choice
BLOCK C: Developing Organizations and People				
*Class 8:* Meaning of work	• Service-learning activities	• Discussion of the latest news • Oral presentation on the play *The Death of a Salesman* • Watching clips from the movie *Up in the Air* • Discussion on the meaning of work	• Reading of the play *The Death of a Salesman*	• Analysis and illustrations of the play *The Death of a Salesman* • Open reflection on a topic of their choice
*Class 9:* Ethical leadership	• Service-learning activities	• Discussion of the latest news • Oral presentation on the movie *Coach Carter* • Conference of Mai Duong, founder of SwabTheWorld	• Watching the movie *Coach Carter*	• Analysis and illustrations of the movie *Coach Carter* • Open reflection on a topic of their choice
*Class 10:* Courage to act	• Service-learning activities	• Discussion of the latest news • Oral presentation on the movie *Erin Brockovich* • Discussion on ethical leadership and the courage to act	• Watching the movie *Erin Brockovich*	• Analysis and illustrations of the movie *Erin Brockovich* • Reflection on the presentation of Mai Duong
*Class 11:* Critical look at Organizations and society	• Service-learning activities	• Discussion of the latest news • Oral presentation on the play *J’aime hydro* • Discussion on ethical leadership	• Listening the play *J’aime hydro* or watching the documentary *Icarus*	• Analysis and illustrations of the movie *Erin Brockovich* • *J’aime hydro* or the documentary *Icarus* • Open reflection on a topic of their choice
*Class 12:* Environmental ethics and conclusion	• Service-learning activities • Visit to the museum			
*Final exam*			• Watching of the documentary *Downfall: The case against Boeing*	• Analysis and illustrations of the museum exposition • Reflection and illustration of the service-learning experience • Analysis and illustrations of the case Boeing 737 Max, based on the documentary *Downfall: The case against Boeing*

*Note*. The color scheme used in Annex 2 and 3 corresponds to the colors used for each learning space (community, classroom, home, portfolio), in line with Figure 1.

**Annex 4.** Grading Rubric Example.

Portfolio section	Weight (points)	Evaluation criteria	Description of criteria
Left page (directed assignment)	2.5	• Relevance and synthesis (0.7 points) • Artwork analysis (0.7 points) • Critical reflection (0.7 points) • Effort / originality (0.4 points)	The entry must demonstrate a clear and relevant synthesis of course concepts, a thoughtful analysis of the assigned artwork, and a critical reflection that connects ethical or organizational themes. Originality and personal engagement are valued.
Right page (open assignment)	2.5	• Description of topic/issue (0.7 points) • Critical reflection (0.7 points) • Connections to course material (0.7 points) • Effort / originality, link between content and artistic presentation (0.4 points)	The entry should show personal critical reflection and explicit links to course materials or themes. The coherence between the topic and the artistic expression is assessed, as well as the student’s effort and creative engagement.

Each week, students complete two portfolio entries—a left-page (directed) and a right-page (open) assignment—each worth 2.5 points for a total of 5 points per week.

## Notes

1. While the present article introduces SpaceJam as an integrative pedagogical strategy, the specific role, functions, and impacts of the artistic portfolio at its core are examined in greater depth in Martineau & Cyr (2025). In that article, we analyze how the portfolio operates as an individual “safe haven” that supports emotional regulation, self-expression, and ethical deliberation in increasingly polarized classroom environments.
